# The use of intravenous Milrinone to treat cerebral vasospasm following traumatic subarachnoid hemorrhage

**DOI:** 10.1186/2193-1801-3-633

**Published:** 2014-10-27

**Authors:** Oliver Lasry, Judith Marcoux

**Affiliations:** Department of Neurology and Neurosurgery, McGill University Health Centre, 1650 Cedar Ave., room L7-516, H3G 1A4 Montreal, QC Canada

**Keywords:** Traumatic brain injury, Subarachnoid hemorrhage, Vasospasm, Milrinone, Post-traumatic vasospasm

## Abstract

**Introduction:**

Traumatic subarachnoid hemorrhage (SAH) is a common intracranial lesion after traumatic brain injury (TBI). As in aneurysmal SAH, cerebral vasospasm is a common cause of secondary brain injury and is associated with the thickness of traumatic SAH. Unfortunately, there is limited literature on an effective treatment of this entity. The vasodilatory and inotropic agent, Milrinone, has been shown to be effective in treating vasospasm following aneurysmal SAH. The authors hypothesized that this agent could be useful and safe in treating vasospasm following tSAH.

**Case descriptions:**

Case reports of 2 TBI cases from a level 1 trauma centre with tSAH and whom developed delayed ischemic neurological deficits (DINDs) are presented. Intravenous Milrinone treatment was provided to each patient following the “Montreal Neurological Hospital Protocol”.

**Discussion and evaluation:**

Both patients had an improvement in their DINDs following the treatment protocol. There were no complications of treatment and the Glasgow Outcome Scores of the patients ranged from 4 to 5.

**Conclusion:**

This is the first report of the use of intravenous Milrinone to treat cerebral vasospasm following traumatic SAH. This treatment option appeared to be safe and potentially useful at treating post-traumatic vasospasm. Prospective studies are necessary to establish Milrinone’s clinical effectiveness in treating this type of cerebral vasospasm.

## Introduction

The most frequent cause for subarachnoid hemorrhage (SAH) is traumatic brain injury (TBI) (Eisenberg et al. 1990). Amongst TBI patients, traumatic subarachnoid hemorrhage (tSAH) is one of the commonest traumatic brain lesions (Chieregato et al. 2005). The literature has described that TBI patients with tSAH have worse outcomes compared to those without this lesion (Kakarieka et al. 1994, 1995). Cerebral vasospasm and delayed ischemia have been shown to be a key process leading to these inferior patient outcomes, which occur in up to 40% of severe TBI patients (Lin et al. 2012; Zurynski and Dorsch 1998). Moreover, the pathophysiology of vasospasm following tSAH is similar to the one occurring after aneurysmal SAH (aSAH) (Wilkins and Odom 1970; Werner and Engelhard 2007). The incidence of cerebral vasospasm after tSAH is strongly related to the thickness of the SAH. In fact, the latter was correlated to the Fisher Classification commonly used to predict the risk of vasospasm after aSAH (Chieregato et al. 2005; Taneda et al. 1996). Despite the similarities between these two subtypes of SAH, there has been limited investigation in cerebral vasospasm occurring after tSAH.

Calcium channel blockers are effective in improving outcomes in TBI patients with tSAH (Langham et al. 2003). Other than this established neuroprotective strategy, patients with tSAH are currently being treated based on data extrapolated from the aSAH literature, which includes the potentially harmful effects of Triple-H Therapy (Zurynski and Dorsch 1998). The unfavorable effects of this therapy in TBI patients relates to the difficulty these patients have in tolerating excessive fluid volume and a low hematocrit. These physiological maneuvers increase the possibility of a secondary brain injury by increasing intracranial pressure and lowering oxygen delivery to the brain. Recently, Lannes et al. (2012) developed “The Montreal Neurological Hospital Protocol” to treat patients with cerebral vasospasm following aSAH. Briefly, their protocol uses intravenous milrinone and the maintenance of homeostasis to control symptomatic vasospasm following aSAH. Given the similarities between the vasospasm occurring after aSAH and tSAH, the authors hypothesized that maintenance of physiologic homeostasis and the concurrent use of intravenous Milrinone, as per the “Montreal Neurological Hospital Protocol”, was a potentially effective, safe and appropriate management strategy for TBI patients who develop cerebral vasospasm from tSAH.

## Case descriptions

We are presenting 2 case reports of patients who presented to a level 1 trauma centre with a diagnosis of tSAH who subsequently developed delayed ischemic deficits during their admission and were thereafter treated with intravenous Milrinone. The severity of the TBI was classified using the GCS. Once delayed ischemic symptoms started, each patient was transferred to the ICU for neuromonitoring and initiation of intravenous Milrinone. They both had a central venous catheter inserted on arrival to the ICU to monitor their volume status with their central venous pressure as a surrogate. “The Montreal Neurological Hospital Protocol” described by Lannes et al. (2012) was followed strictly in the treatment of all patients included in this case report. The protocol initially focuses on excluding other etiologies for the delayed ischemic deficits such as metabolic/electrolyte abnormalities, hydrocephalus, recurrent hemorrhage, seizures or infection. Thereafter, a plain CT of the head is performed. Once these investigations reveal no other cause for the neurological deterioration, radiological confirmation of the vasospasm with either transcranial doppler, CTA or DSA is completed. The first Milrinone bolus is then administered (0.1 mg/kg) and the intensive care team ensures that the patients’ deficits improve or revert before starting a maintenance infusion (0.75 μg/kg/min). Normal saline boluses (0.9% NS) are used for any patients who have a central venous pressure below 6 mmHg. The use of intravenous norepinephrine is only employed if the patient’s blood pressure falls below their baseline, as recorded during their admission before being transferred to the ICU. Once the patients have their delayed ischemic deficits reversed/improved for at least 48 hours, weaning of the Milrinone infusion is started by reducing the dose by 0.25 μg/kg/min decrements. If there are any recurrent DINDs, the patients are placed back on the dose they were previously receiving. If required, another Milrinone bolus is administered if the patient’s deficits do not revert. At our institution, all TBI patients who have a tSAH with Fisher Grade 3 or above are treated with Nimodipine (60 mg orally once daily for 21 days) (Harders et al. 1996; Rosen and Macdonal 2005). Therefore, Nimodipine treatment was part of the treatment protocol for all the cases presented. In addition, each patient had either a CTA or MR-Angiography of the brain, at baseline, to exclude a causative vascular lesion for the SAH. No patient in this report had such lesion. The 6-month functional outcome of patients was determined using the GOS. Table 1 summarizes the clinical evolution of the 2 patients included in this report. As per institutional guidelines, the charts review and reports are approved by the Director of Professional Services.

**Table 1:** **Summary of case reports**

**Case number**	1	2
**Age (years)**	64	64
**Medical history**	Cerebral abscess, epilepsy	Alcoholism
**Mechanism of injury**	Fall from own height	Fall from own height
**Initial GCS**	14	14
**Initial CT-Head finding**	SAH in left sylvian fissure	Diffuse SAH, concentrated in left sylvian fissure
**Fisher grade of SAH**	3	3
**Onset of DINDs (after admission)**	8	7
**Presenting symptoms**	Aphasia, right hemiparesis	Aphasia, dysarthria, lethargy, right hemiparesis
**Imaging modality to investigate vasospasm**	DSA	CTA
**Duration of Milrinone treatment/ICU stay (days)**	9	10
**Recurrence of DINDs**	No	Yes
**GOS at 6 months**	4	5

### Case 1

#### History

The first case is a 64 year-old male who was previously known for cerebral abscess, which resulted in chronic epilepsy. His medication profile consisted only of Carbamazepine. He presented to our trauma centre after having a generalized tonic-clonic seizure resulting in a witnessed fall from his own height.

#### Examination and clinical course

**Figure 1:**
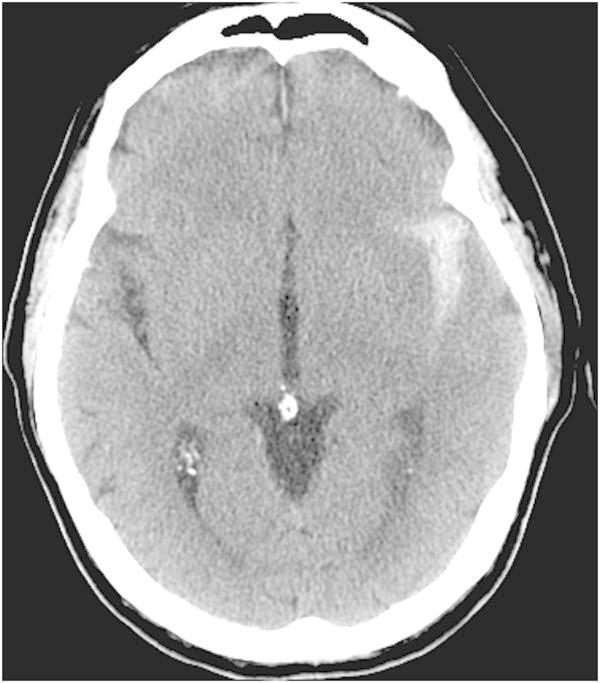
**Initial CT-Head reveals SAH concentrated in the left sylvian fissure with a thickness of > 1.0 mm (Fisher Grade 3).**

**Figure 2:**
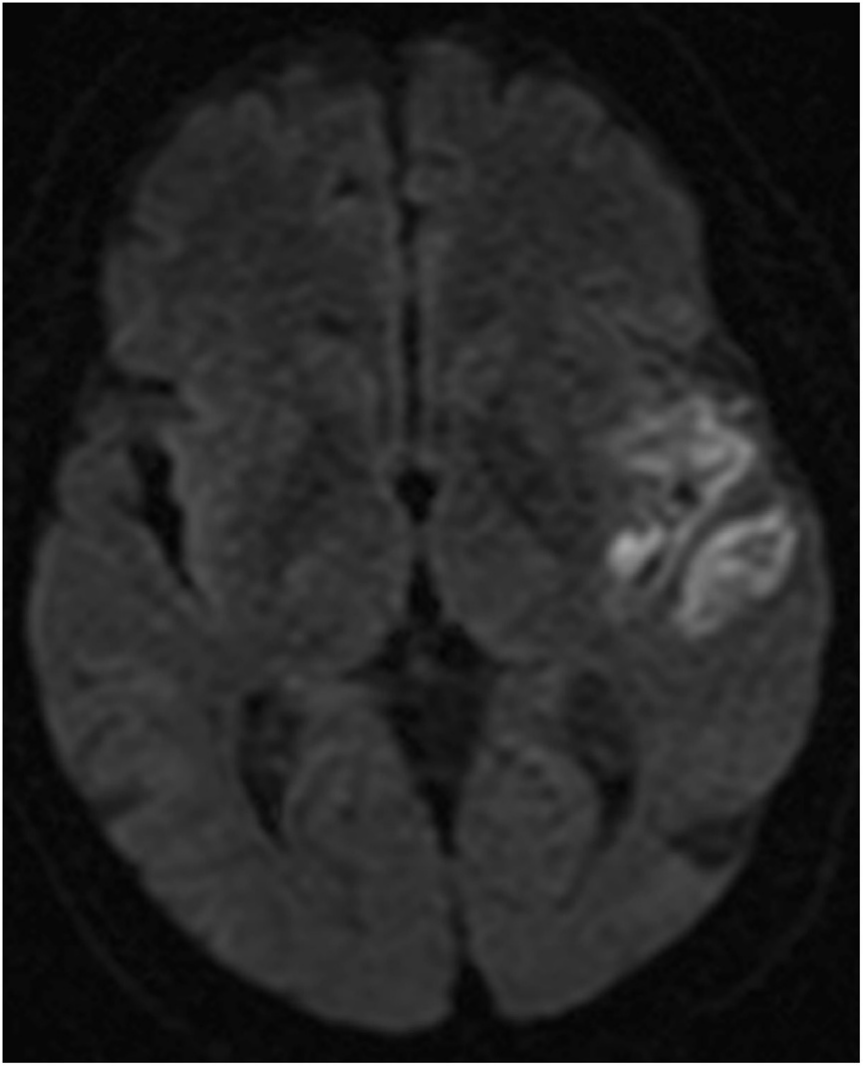
**DWI MRI sequence completed once the patient presented with delayed ischemic symptoms.** There is evidence of restricted diffusion in DWI with hyperintensity in the ADC map (not shown) in the same left middle cerebral artery vascular territory, indicating early subacute infarction.

**Figure 3:**
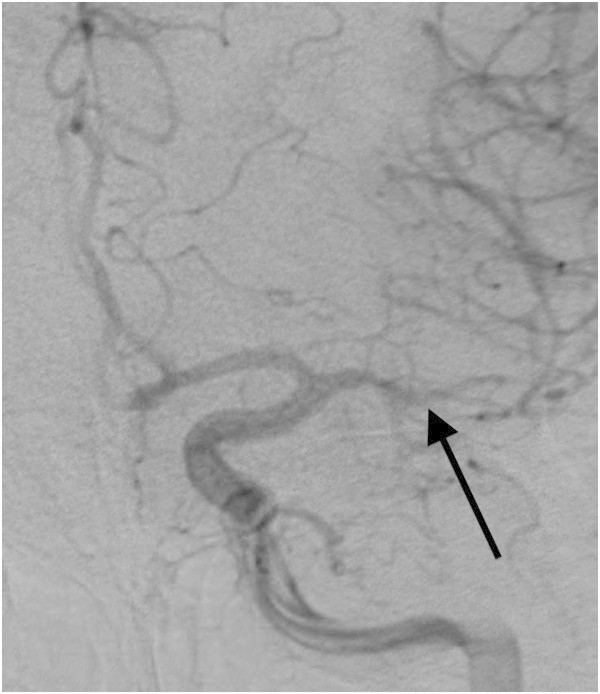
**DSA confirming vasospasm in the narrowed M1 and M2 segments (indicated by the arrow) of the left middle cerebral artery.**

On arrival, his Glasgow Coma Score (GCS) (Teasdale and Jennett 1974) was 14 with disorientation to time and place. The rest of his neurological exam was non-localizing and his hemodynamic status was stable. His initial computed tomography (CT) of the head revealed a thick collection of subarachnoid hemorrhage localized to the left sylvian fissure (Figure 1). During the first 3 days of admission his neurological status had normalized. Eight days after the trauma, the patient’s wife noted that his neurological status had changed. She therefore brought him back to the emergency room where he was found to have a right-sided hemiparesis, a right lower facial droop, dysarthria and global aphasia. He was immediately admitted to the Intensive Care Unit (ICU) for neuro-monitoring. A magnetic resonance imaging of the brain revealed restricted diffusion in the left MCA territory consistent with a subacute infarct (Figure 2). Thereafter, the Milrinone protocol was started and within 90 minutes, the patient’s symptoms had improved. The patient had a formal (Digital Subtraction Angiography) DSA that confirmed vasospasm of the M1 and M2 segments of the left middle cerebral artery (MCA) (Figure 3). The protocol was continued for a total of 9 days and the weaning of Milrinone started on the 5^th^ day. The patient was discharged from hospital with a slight expressive aphasia and right hemiparesis, which was improved from his initial presentation.

### Case 2

#### History

The second case is a 64 year-old female, with a past medical history of alcoholism, who presented to our emergency department, intoxicated with alcohol, after falling from her own height.

#### Examination and clinical course

**Figure 4:**
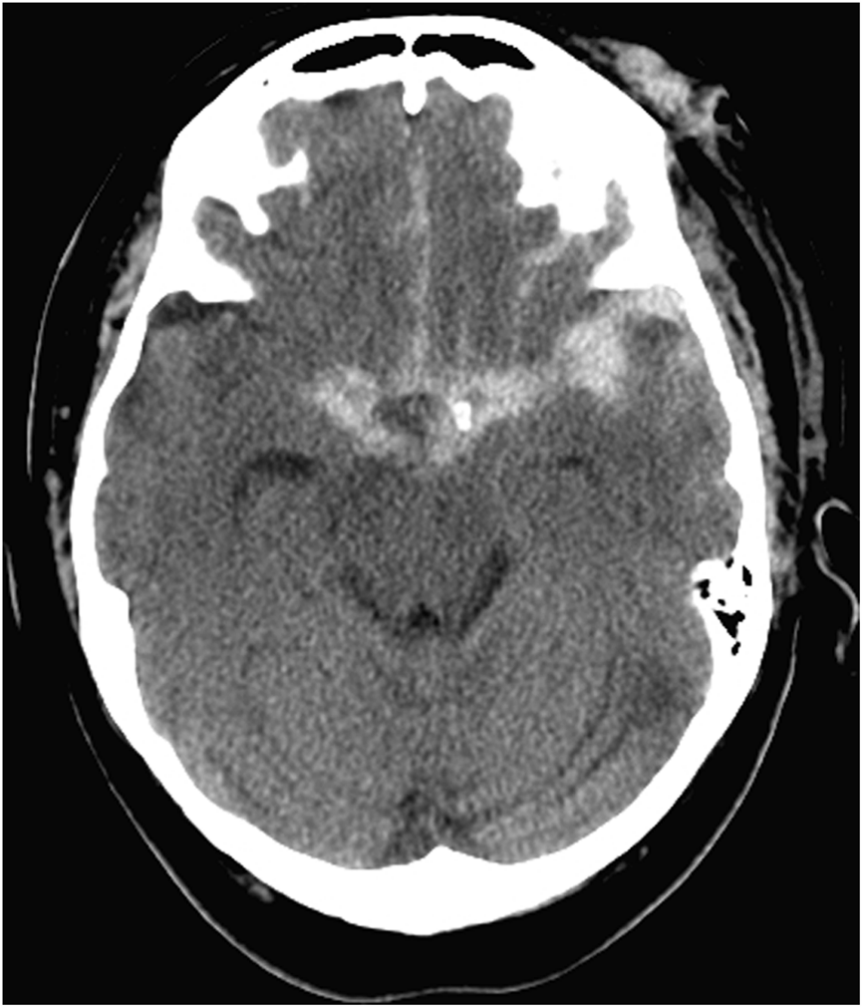
**CT-Head demonstrating diffuse subarachnoid hemorrhage in bilateral basal cisterns which is mainly concentrated in the left sylvian fissure (Fisher Grade 3).**

On arrival, her vital signs were stable and she had a GCS of 14. A CT-head was completed which revealed diffuse and thick subarachnoid hemorrhage, which was mainly centered over the left sylvian fissure and convexity (Figure 4). The patient’s GCS was 15 without any neurological deficits within 36 hours of her admission. On the 7^th^ day post-trauma, she was found to be somnolent, expressively aphasic, dysarthric and weak on her right hemibody with a right lower facial droop. She was transferred to the ICU and had a CT-Angiography (CTA) that revealed focal narrowing of the M1 and M2 segments of the left MCA. She was therefore started on the Milrinone protocol. Her delayed ischemic deficits completely resolved within 2 hours. Weaning of the drug started 4 days after her ICU admission. She was slowly weaned off Milrinone over 6 days due to recurrence of DINDs. She had no residual deficits after her discharge from the ICU.

## Discussion and evaluation

Historically, the literature on the identification and treatment of cerebral vasospasm after tSAH has been limited. In the last two decades, there has been a significant rise in the description of this phenomenon. Despite these advances, the literature’s recommendations on the management of these patients are based on extrapolating data from the literature of vasospasm after aSAH. This type of approach is intuitively appealing because of the similarities in pathophysiology that have been shown between the two entities (Wilkins and Odom 1970; Werner and Engelhard 2007). tSAH thickness and severity has been consistently shown to be a risk factor for the development of cerebral vasospasm after TBIs (Zubkov et al. 2000). The development of delayed ischemia in these last patients has been shown to be almost identical to patients who suffered an aSAH. Taneda et al. showed that patients with higher-grade tSAH on the Modified Fisher Scale had an increased risk of vasospasm and delayed ischemia (Taneda et al. 1996). These episodes peaked between post-trauma days 4 to 16, just as described in aSAH. However, there are subtle differences between the two processes. TCD studies have revealed that patients with TBI develop cerebral vasospasm earlier than patients with aSAH (Taneda et al. 1996; Lee et al. 1997; Oertel et al. 2005; Servadei et al. 2002). This earlier onset, usually within 48 hours, is explained by the fact that TBI patients have traumatic forces applied to their cerebral vessels at the time of injury and a release of vasoactive substance from injured brain that can induce earlier vasospasm (Zurynski and Dorsch 1998). Furthermore, predicting which patients with tSAH will develop cerebral vasospasm is still not established. Studies investigating tSAH have shown that this lesion predicts worse outcomes but that it may be related to a multitude of causes, such as: progression of other traumatic hematomas, older age and the severity of the initial injury (Chieregato et al. 2005; Servadei et al. 2002). Evidently, there is still a significant lack of knowledge pertaining to the pathophysiology and natural history of cerebral vasospasm after tSAH. Still, there is enough evidence supporting that cerebral vasospasm can contribute to secondary brain injury in TBI patients and that treatment measures should be sought.

As mentioned earlier, the treatment of this vasospasm has been undertaken by implementing similar strategies as in aSAH. A Cochrane Review of six randomized-controlled trials on the use of Nimodipine in TBI showed that the subgroup of patients with tSAH had a decreased risk of death and severe disability compared to placebo (Langham et al. 2003). There was the added caution of an increased risk of hypotension in these treated patients that may worsen outcome. Our 2 patients were treated with Nimodipine. They did not develop hypotension, yet Nimodipine was not sufficient to prevent clinically significant vasospasm. In contrast, traditional treatment with Triple-H therapy has been tried in the tSAH population with poor results and treatment efficacy (Zurynski and Dorsch 1998; Oertel et al. 2005). Furthermore, TBI patients may have other traumatic lesion such as contusions, which may be at-risk of progressing if hypertensive therapy is started. Also, hypervolemia can worsen cases of post-traumatic cerebral edema (Oertel et al. 2005). There have been case reports of using endovascular therapies for cerebral vasospasm after tSAH. Intra-arterial papaverine therapy and balloon dilatation angioplasty were somewhat successful in these instances, but come with the risk of an endovascular approach such as ischemic stroke (Newell et al. 1992; Vardiman et al. 1995). Despite having described adequate neuroprotective measures for vasospasm after tSAH, the literature has not described any other effective treatments to reverse this clinical entity. Milrinone is a potent phosphodiesterase Type III inhibitor with both inotropic and vasodilatory properties and perhaps anti-inflammatory properties at the level of the cerebral vessel’s wall (Lannes et al. 2012, Vardiman et al. 1995; Hayashida et al. 1999; Vroom et al. 1996). It has a concentration-dependant effect and can achieve significant vasodilatation at sufficient concentration (Nishiguchi et al., 2010). Given the knowledge of the pathophysiology of vasospasm after tSAH mentioned previously, Milrinone appears to be a suitable therapy for this patient population. The “Montreal Neurological Hospital Protocol’s use of homeostasis and Milrinone avoids all of the potential complications that Triple-H therapy can cause in TBI patients. In addition, this type of approach most often avoids the need for any endovascular therapy that requires a potentially unstable patient to leave the ICU (Lannes et al. 2012). In the two patients we treated, there were no complications of treatment cases and all patients had good neurological outcomes based on their 6-month Glasgow Outcome Scale (GOS) (Jennett and Bond 1975).

Trauma centers have not uniformly adopted protocols for the detection of vasospasm in patients with TBI. The most recent Brain Trauma Foundation Guidelines do not refer to this potentially treatable pathology (Bratton et al. 2007). The literature has referred to using TCD, CTA and DSA (Zurynski and Dorsch 1998). Neurological examination may be a reliable indicator as to when delayed ischemic symptoms begin in patients with mild TBI. In cases of moderate to severe TBI patients, this type of examination may be impossible because the patient’s neurological status is severely compromised. The clinician may therefore miss subtle deficits that may occur with vasospasm and delayed ischemia. For this reason, surveillance protocols with radiological exams and/or electro-encephalographic monitoring are of utmost importance for detecting vasospasm in these patients. Unfortunately, there is no existing consensus in the literature regarding a specific protocol to follow.

There are limitations to extrapolating any conclusions based on these case reports. As Lannes et al. mention, there are many factors that can cause symptoms similar to DINDs in the context of SAH (Lannes et al. 2012). We attempted to exclude these other causes by completing the appropriate biochemical and imaging investigations. Despite these limitations, we believe that in mild TBI patients, clinical examinations can be quite informative in terms of formulating a diagnosis of cerebral vasospasm. Further studies are necessary to quantify the beneficial effect of Milrinone on cerebral vasospasm after tSAH, especially in heterogeneous populations that include mild to severe TBI cases, as well as both surgically and medically treated cases.

## Conclusions

This report served to describe a possible treatment avenue for cerebral vasospasm after tSAH using intravenous Milrinone and homeostatic maneuvers, which differs from what is currently offered in the literature. This treatment modality appeared safe for TBI patients. It also was effective in conjunction with Nimodipine for our patients. Further prospective studies investigating appropriate surveillance protocols for patients with TBI who are at-risk for developing vasospasm are necessary. Only then can further studies investigating the use of Milrinone, in addition to other possible treatment measures, be completed to establish the management of this type of secondary brain injury.

## Consent

The written consent was obtained from one of the patients for the publication of this report and any accompanying images. For the other patient, the written consent was obtain form the patient’s next of kin (the patient has since died of unrelated cause).

## Abbreviations

aSAH: Aneurysmal subarachnoid hemorrhage
CT: Computed tomography
CTA: Computed tomography angiogram
DINDs: Delayed ischemic neurological deficits
DSA: Digital substraction angiography
GCS: Glasgow coma scale
GOS: Glasgow outcome scale
ICA: Internal carotid artery
ICP: Intracranial pressure
ICU: Intensive care unit
MCA: Middle cerebral artery
NS: Normal saline
SAH: Subarachnoid hemorrhage
TBI: Traumatic brain injury
TCD: Transcranial Doppler
tSAH: Traumatic subarachnoid hemorrhage.
